# Nanopesticides: A Systematic Review of Their Prospects With Special Reference to Tea Pest Management

**DOI:** 10.3389/fnut.2021.686131

**Published:** 2021-08-10

**Authors:** Bhabesh Deka, Azariah Babu, Chittaranjan Baruah, Manash Barthakur

**Affiliations:** ^1^North Bengal Regional Research and Development Centre, Nagrakata, India; ^2^Postgraduate Department of Zoology, Darrang College (Affiliated to Gauhati University), Tezpur, India; ^3^Department of Zoology, Pub Kamrup College, Baihata Chariali, India

**Keywords:** nanotechnology, tea, insect pest, nano pesticides, IPM

## Abstract

**Background:** Tea is a natural beverage made from the tender leaves of the tea plant (*Camellia sinensis* Kuntze). Being of a perennial and monoculture nature in terms of its cultivation system, it provides a stable micro-climate for various insect pests, which cause substantial loss of crop. With the escalating cost of insect pest management and increasing concern about the adverse effects of the pesticide residues in manufactured tea, there is an urgent need to explore other avenues for pest management strategies.

**Aim:** Integrated pest management (IPM) in tea invites an multidisciplinary approach owing to the high pest diversity in the perennial tea plantation system. In this review, we have highlighted current developments of nanotechnology for crop protection and the prospects of nanoparticles (NPs) in plant protection, emphasizing the control of different major pests of tea plantations.

**Methods:** A literature search was performed using the ScienceDirect, Web of Science, Pubmed, and Google Scholar search engines with the following terms: nanotechnology, nanopesticides, tea, and insect pest. An article search concentrated on developments after 1988.

**Results:** We have described the impact of various pests in tea production and innovative approaches on the use of various biosynthesized and syntheric nanopesticides against specific insect pest targets. Simultaneously, we have provided support for NP-based technology and their different categories that are currently employed for the management of pests in different agro-ecosystems. Besides the broad categories of active ingredients (AI) of synthetic insecticides, pheromones and natural resource-based molecules have pesticidal activity and can also be used with NPs as a carriers as alternatives to traditional pest control agents. Finally, the merits and demerits of incorporating NP-based nanopesticides are also illustrated.

**Conclusions:** Nanopesticides for plant protection is an emerging research field, and it offers new methods to design active ingredients amid nanoscale dimensions. Nanopesticide-based formulations have a potential and bright future for the development of more effective and safer pesticide/biopesticides.

## Highlights

- Applications of nanoparticles (NPs) in plant protection, emphasizing the control of different major pests of tea plantations
- Categorization of NPs-based technology that is currently employed for management of pests in different agro-ecosystems
- The merits and demerits of incorporating NP-based nanopesticides

## Introduction

### Rationale

**Figure 1:**
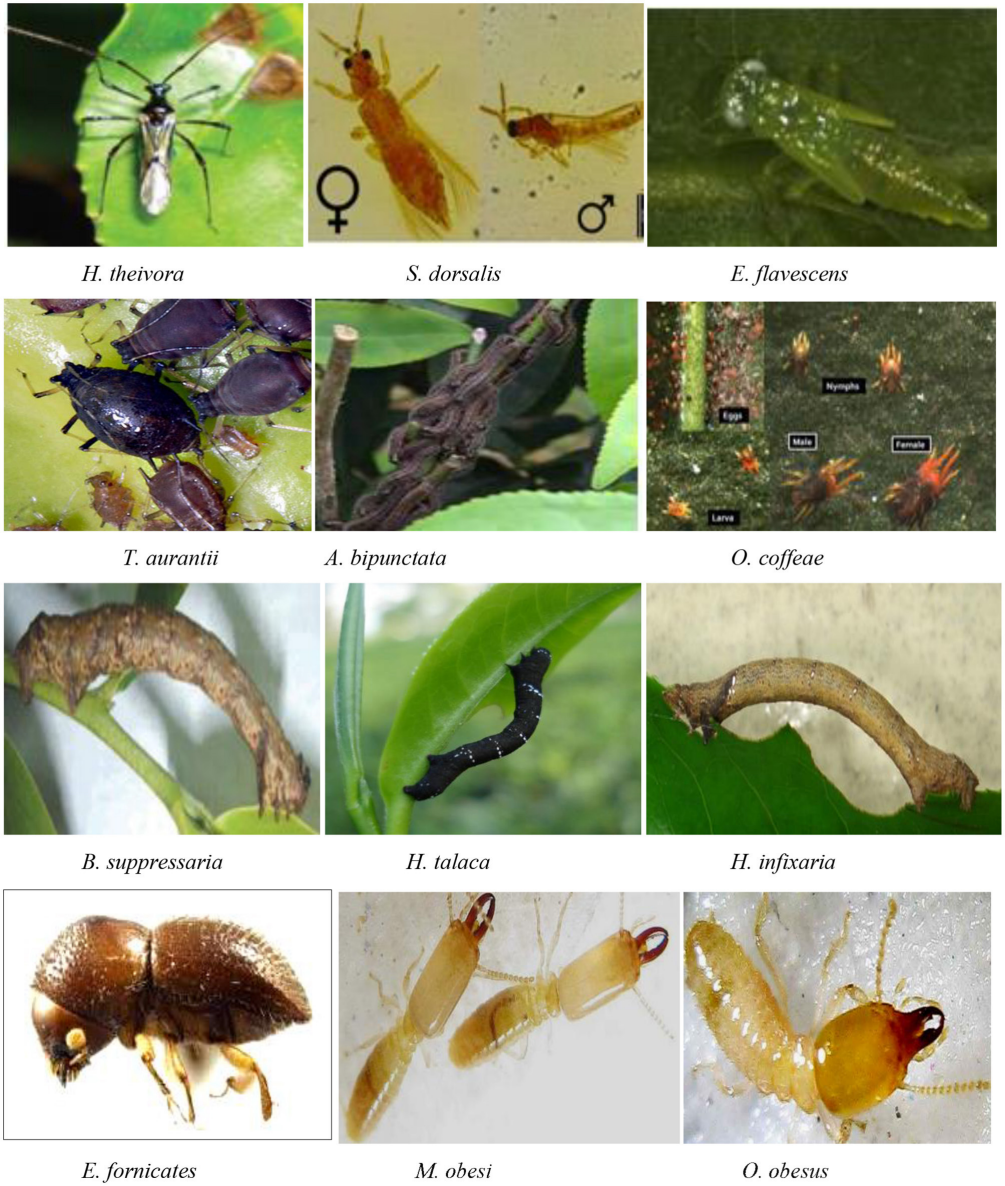
Major pests of tea crop.

After water, tea leaves (*Camellia sinensis*) are used to form the most extensively consumed drink on the planet (1). Tea drinking has been shown to have numerous health benefits, including antioxidant, anti-obesity, and anticancer properties. As a result, researchers have paid close attention to tea phytochemicals in terms of structure confirmation, formation mechanism, component clarification, and bioactivity screening of potential constituents (2). Tea is high in bioactives and has been shown to have antiviral properties. Green tea catechins (GTCs) are polyphenolic chemicals found in *C. sinensis* leaves (3). Green tea drinking has been linked to a reduction in the risk of carcinogenesis in a variety of cancers, including prostate cancer (4). Black tea constituents theaflavin 3,3'-digallate, theaflavin 3-gallate, and procyanidin B2 have the potential to serve as inhibitors for SARS-CoV-2 targets and could be used as therapeutic candidates in future investigations against COVID-19 (5). As a result, the safety and quality of raw tea leaves are critical indicators in the production of tea and related products (1). However, the tea plantations provide a stable microclimate and food supply for several notorious pests such as insects, mites and nematodes, etc., (Table 1 and Figure 1) due to the perennial and monoculture nature of tea plant cultivation, which causes substantial crop loss annually. However, geographical variation in pest diversity exists due to the effect of climate change, altitude, the age of the plantation, etc.

**Table 1:** Different pests of tea crop [source: Deka et al. (6, 7); Babu (8); Hazarika et al. (9)].

**Common name**	**Scientific name**
**Major pests of tea**	
Tea mosquito bug:	*Helopeltis theivora* Waterhouse (Miridae: Hemiptera)
Thrips	*Scirtothrips dorsalis* Hood (Thripidae: Thysanoptera)
Jassid	*Empoasca flavescens* Fab. (Cicadellidae: Hemiptera)
Aphids	*Toxoptera aurantii* Boyer de Fonscolombe (Aphididae: Hemiptera)
Bunch caterpillar:	*Andraca bipunctata* Walker (Bombycidae: Lepidoptera)
Red spider mite	*Oligonychus coffeae* Nietner (Tetranychidae: Acari)
Tea looper complex	*Buzura suppressaria* Guen (Geometridae: Lepidoptera), *Hyposidra talaca* (Walker), *H. infixaria* (Walker) (Geometridae: Lepidoptera)
Shot hole borer	*Euwallacea fornicates* Eichhoff (Scolytidae: Coleoptera)
Live wood eating termite	*Microtermes obesi* (Isoptera:Termitidae)
Scavenging termites	*Odontotermes obesus* (Isoptera:Termitidae)
**Minor pests of tea**	
Flush worm	*Cydia leucostoma* Meyrick (Tortricidae: Lepidoptera)
Pink and Purple mite	*Acaphylla theae* Watt and *Calacarus carinatus* Green (Eriophyidae: Acarina)
Scarlet mite	*Brevipalpus phoenicis* Geijskes (Tenuipalpidae:Acarina)
Yellow mite	*Polyphagotarsonemus latus* Banks (Tarsonemidae: Acarina)
Leaf roller	*Caloptilia theivora* Walsingham (Gracillariidae: Lepidoptera)
Scales	*Saissetia formicarii* Takahashi *S*. *coffeae* Walker*, Eriochiton theae* Green*, Coccus viridis* Green (Coccidae: Hemiptera)
Tea tortrix	*Homona coffearia* Nietner (Tortricide: Lepidoptera)

Even though there are numerous non-conventional methods available, pest control in the tea ecosystem is based on the use of synthetic pesticides. Although ATP (adenosine triphosphate) disruptors of IGR (insect growth regulators) have been introduced recently for insect pest management, neurotoxic pesticides (mode of action that interferes the nervous system of insects) are still in use for the management of insect pests, which poses a risk to mammals and non-target organisms besides having an environmental impact (10). In addition to this bioaccumulation, environmental contamination, and mammalian toxicity, continuous application of these pesticide compounds accounts for major key challenges in agriculture due to resistance development in insect pests. Therefore, novel and innovative strategies using advanced technologies are essential to address those problems. Nanotechnology, a promising and emerging research field, could help towards attaining these goals for crop protection as it offers new methods for designing novel AIs using existing pesticides with nanoscale dimensions (below 1,000 nm) along with the target-specific delivery of the nano-emulsions, dispersion, and formulation, and these can be referred to as nanopesticides (11–13). Nanopesticides are expected to deal with the limitations of existing strategies to control insect pests and provide newer and advanced formulations that can penetrate the insect body, remain active and stable in the target ecosystem and be benign to the non-target organism, be cost-effective, and minimize defense of the target pests (11, 14, 15).

### Objectives

The aim of this article was to look at how nanotechnology is being used in crop protection now and in the future. It examined nanoscientific developments of potential nanoparticles (NPs) for agricultural pest control with a focus on the control of various major insect pests of tea plantations using nanoparticle-based pesticide delivery systems, polymer nanocarriers, inorganic nanocarriers, mineral-based nanoparticles, and Nano bio-pesticides. We have also conducted a brief review of the literature on nanoscientific advances in nanotechnology, nanopesticides, and integrated insect pest management in tea plantations.

## Methods

A comprehensive literature search was conducted using Web of Science, ScienceDirect Pubmed, Google Scholar, and other online sources. The article search focused on nanotechnology, nanopesticides, tea, and insect pests in the domain of “Agricultural and Biological Sciences” in the subdomains of Agricultural and Biological Sciences (general) and Agronomy and Crop Sciences." Original articles from 1988 onwards were of great interest. The research experience of the authors as well as information gleaned through personal communication are also included.

## Results and Discussion

### Results of the Literature Search and Selection

**Figure 2:**
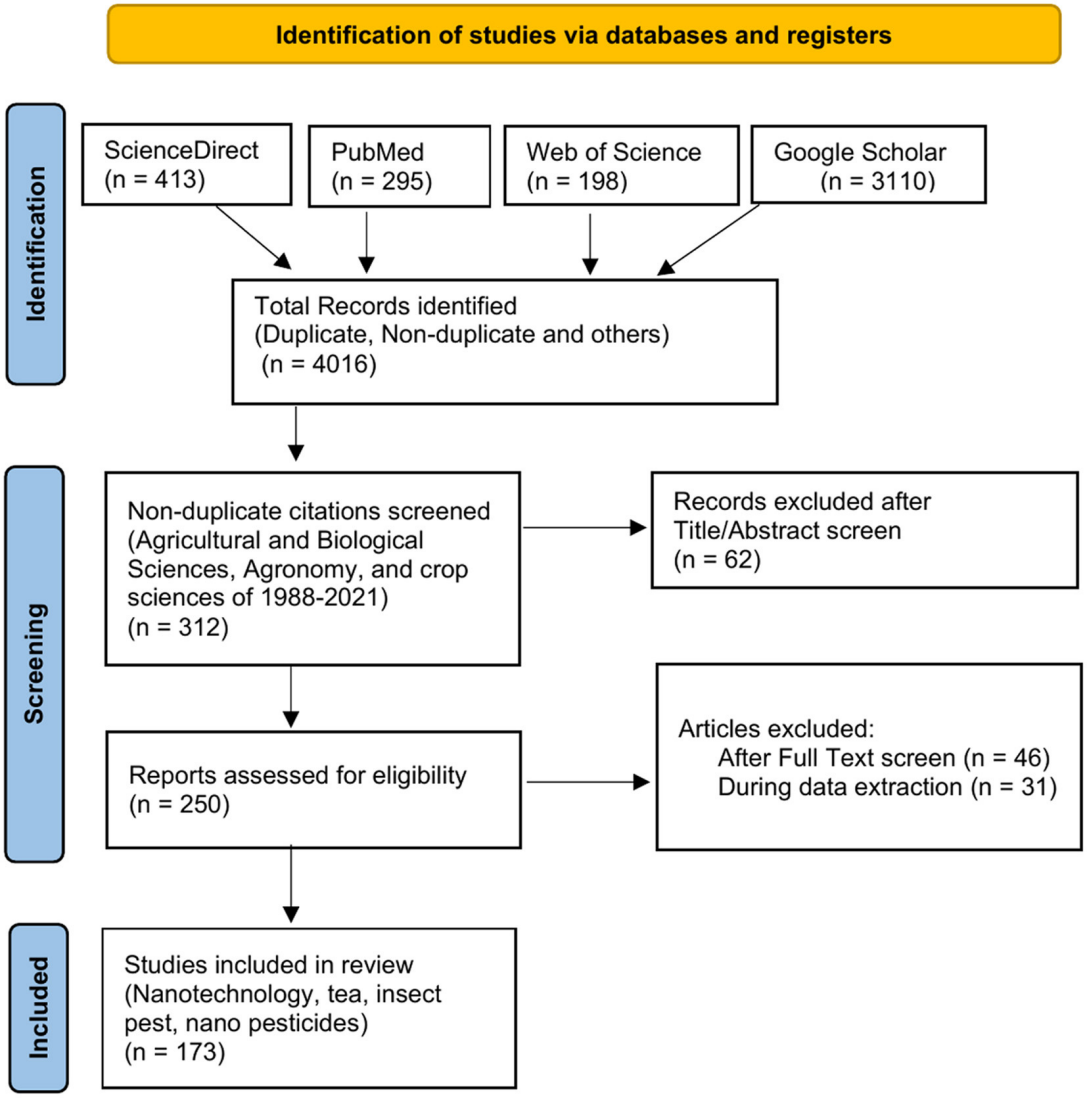
The flowchart of the literature search, screening and selection.

A total of 312 non-duplicate publications were obtained from the four databases, with 62 articles being eliminated after the Title/Abstract Screen. In total, 250 papers were obtained for full-text evaluation after 62 duplicates were removed and title/abstract screening was performed. Following a review of these full-text publications, 46 articles were found to be excluded after Full-Text Screening and 31 articles were found to be Excluded During Data Extraction. Finally, the analysis comprised 173 publications on Nanotechnology, tea, insect pests, and nanopesticides. Figure 2 depicts the screening and selection method.

### Impact of Pests on Tea Production

Tea, as a perennial mono-crop cultivated in favorable climatic conditions, has become conducive for a large number of insects and mite pests, disease, and weeds as a food source, and this needs to be managed to avoid huge crop losses. More than 1,030 species of arthropods are linked to the tea ecosystem throughout the world, and due to various pests, the entire tea industry has seen a crop loss of 20–35% despite using plant protection formulations. From root to tender shoots, all parts of the tea bush are attacked by various pest species (9).

**Figure 3:**
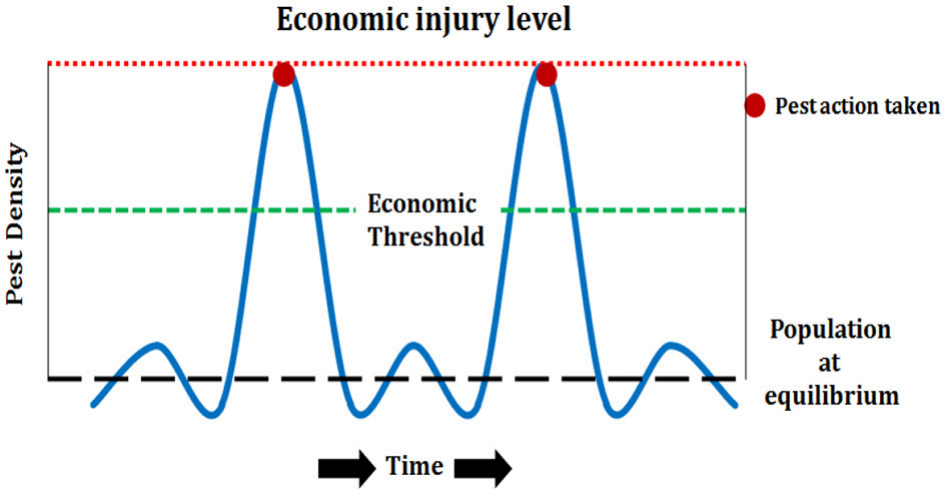
Economic injury level (EIL) and Economic Threshold Level (ETL) of tea pests.

To take appropriate control measures, the economic threshold level (ETL) is considered as one of the crucial requirements, and it indicates the density of population at which different control measures for the management of pests should be adopted to avert increasing pest population from touching the economic injury level (EIL) (Figure 3 and Table 2). In tea plantations, the EIL is very high for those pests that infest the new shoots as their damage directly affects the yield, and in cases for the pests that infest the other parts of the tea bush, the immediate EIL is low, but their damage affects the growth of the new shoots as well as the health of the bush (17).

**Table 2:** Economic Threshold Level (ETL) of major pests of tea (16).

**Name of the pest**	**Economic threshold level (ETL)**
Tea Mosquito Bug	5% infestation
Aphids	20% infestation
Thrips	3 Thrips per shoot
Jassids	5 jassids per shoot
Looper caterpillar	4–5 Lopper per plant
Flushworm, Leaf Rollers	5 infested rolls per bush
Red Spider Mites, Pink and Purple Mites	4 mites per leaf
Termites	10% infestation
Nematodes	6 numbers of nematode/10 gm of soil

### Management of Tea Pests in Conventional Methods

**Figure 4:**
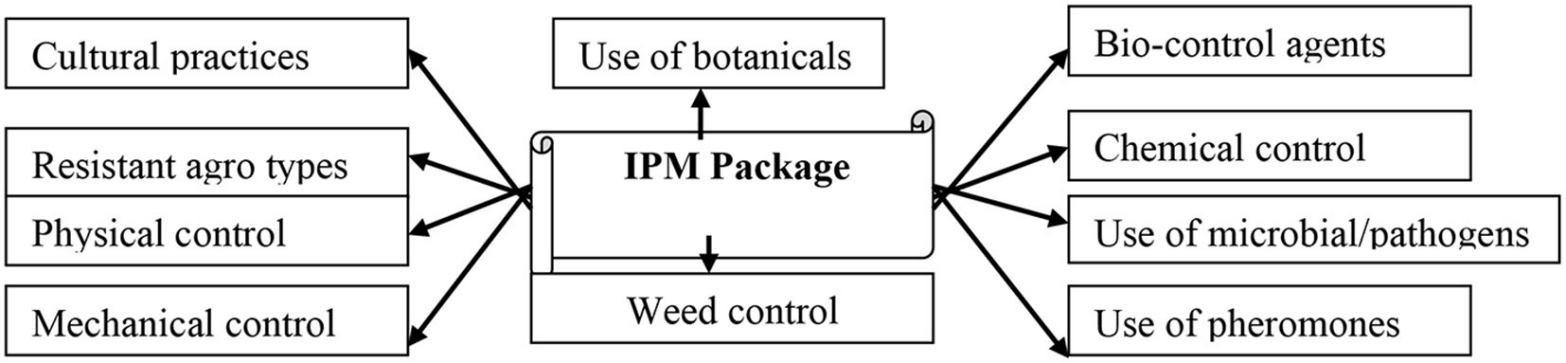
IPM components for tea pests.

Based on the ecological characteristics of tea fields and the cropping system of tea, a tentative IPM system comprising all possible control methods has been proposed (Figure 4) (6, 8, 17).

### Biological Control

The three common approaches followed for biological control of tea pests include classical, conservation, and augmentative biological control. More than 170 species of parasites, entomopathogens (entomopathogenic fungi, entomopathogenic nematodes, viruses, and bacteria), predators (coccinellids, syrphids, mirids, phytoseiids, and spiders), parasitoids (braconids, bethylids, eulophids, ichneumonids, tachinids, and muscids), and hyperparasites are reported from typical monoculture tea ecosystems, which are extremely efficient at regulating several insect pests (7, 18–21).

Microorganisms belonging to fungi like *Metarhizium anisopliae, Verticillium lecanii, Steinernema* sp., *Beauveria bassiana*, and *Paecilomyces fumosoroseus* along with baculoviruses *viz*., nuclear polyhedrosis virus (NPV), granulosis virus (GV), etc. occur naturally and are identified and formulated into microbial pesticides by following appropriate techniques for production, standardization, formulation, and application. This has progressed well in India, Japan, Sri Lanka, and China to control the tea pests (22–26). In the cultivation of organic tea, botanicals having pesticidal properties play an important role. Hundreds of botanicals, which are having significant oviposition deterrence or antifeedant or toxic effects, have been reported (6, 27, 28).

### Chemical Control

Insecticides are one of the key strategies to control the tea pests, which includes synthetic pyrethroids, neonicotinoids, spinosyns, avermectins, pyrazoles, and oxidizes (29, 30).

Besides the biological and chemical approaches, several other approaches have been followed for integrated pest management in tea plantations. Cultural practices include pruning and plucking operations, field sanitation and crop refuse destruction, tillage of soils, soil amendments, and fertilizers, trap crops and shade trees, water management, and adopting various mechanical and physical methods *viz*., hand collection and destruction, use of physical or chemical barriers, light traps, attractants, repellents, etc.

### Why Use Nanopesticides for Insect Pest Management in Tea Crop?

Nanopesticides refer to the utilization of nanotechnology for plant protection, an emerging research field, offers new methods to design active ingredients amid nanoscale dimensions, in addition to their formulation and delivery. This field comprises broad research aspects including the study of basic understanding of the interface between insects and nanoscale materials, formulation of AI into nano-emulsions and dispersions with accessible pesticides, preparation of new nano-pesticide using nanomaterials as active pesticide agents or nanocarriers for their delivery (11–13). Extensive research on nanopesticides is expected to deal with the restrictions of the accessible strategies used for pest control and endowed with nano-based novel formulations that enter into the target (pest), remain steady and active in the environment, without impact on non-target organisms, using cost-effective formulation (11, 15).

Auffan et al. (31) defined nanoparticle (NP) as ultrafine particles comprising dimensions from 1 to 100 nm in size and have characteristics that are not shared by non-nano scale particles with a similar chemical composition. The key parameters for the NP for use as nanopesticides include some specific properties *viz*., chemical composition (organic, inorganic, metallic, polymeric, and carbon), shape (rods, spherical, irregular, and tube), the surface to volume ratio, size, and crystal phase (amorphous and crystalline) besides the toxicity. NP-based pesticides tend to show several benefits in comparison to conventional pesticides *viz*. increased solubility in water and the stability of the formulation, its ability to eliminate toxic organic solvents and release the AI at a slower rate, its enhanced mobility, and its insecticidal activity (32). Recent studies indicated that to formulate NP, varieties of materials were synthesized in various forms of chemical compositions including carbon, silicates, polymers, metal oxides, lipids, semiconductor quantum dots, ceramics, emulsions, proteins, and dendrimers (33–35).

### NPs to Improve Pesticide Formulations to Control Insect Pest

Recent studies also showed different plant-synthesized NPs and their efficacy against a wide range of economically important insect pests, including mosquitoes (15), beetles (36), moths (37), hard ticks (38), lice (39), louse flies (40), etc. Hence, most of the formulation of pesticides are available in oil-in-water (O/W) emulsions or emulsifiable concentrates (ECs) and are sparingly water-soluble (41). Usually, EC formulations contain very expensive organic solvents that are more toxic and flammable or a mixture of surfactant emulsifiers. On the other hand, the O/W emulsions are based on a blend of non-ionic and polymeric surfactants, which requires high-energy input for emulsification (42, 43). To address the disadvantages of these conventional pesticides, new formulations are being introduced based on micro and nanoemulsions (41, 44–47). For the management of a wide range of pests and diseases in different agroecosystems, several formulations based on micro-emulsion are available, including systemic fungicides and plant growth regulators (46, 48, 49). Micro-emulsions are steadier than the nano-emulsions (nano-emulsions require high energy input for scale-up for commercial production and are difficult for onsite production by the handlers) and advantageous over the conventional formulations (they have improved stability and tank mix compatibility, reduce toxicity to the handler and flammability, and have increased efficacy due to better uptake and penetration from surfactants having high solubilizing power) (41, 50). Conversely, micro-emulsions showed various disadvantages *viz*., higher surfactant concentrations, with very low AI contents and the limited number of suitable surfactants (45). These limitations could be partly sorted out by different formulation concepts based on nano-suspension or nano-dispersion, where AI of nanocrystals creates nano-dispersions containing similar properties of solutions, and the development of such suitable release systems could enhance the pesticide's efficiency and performance, reducing adverse environmental effects (51). In general, NPs are customized to carry a specific biomolecule to the organ, tissue, or even cell, and using a nanocarrier can easily penetrate the insect or plant cell and accurately deliver the products (52). For delivery and tracking purposes, various inorganic nanomaterials with distinctive chemical and physical properties, *viz*., silica, metal oxides, metals, semiconductors, and carbon-based materials, have been prepared (53). In addition to protecting crop plants from pests and diseases, nano-delivery vehicles can enhance plant growth, yield, seed vigor, and could even be used for manipulation of genes (54). By creating new pores either through ion channels or by binding to a carrier protein, the NPs enter into the plant cells and can cause significant changes to these systems (55). Despite the remarkable capability of the plant cell wall during the entry of NPs (12, 56), some unlikely impact of NP application has been raised, and studies are being continued focusing on phytotoxic effects of NPs (57, 58) and their influence on plant development (59, 60). Electron microscopic studies of the effect of NPs on insects and plants also revealed that they can penetrate the cell, mitochondria, or nucleus of insects and plant easily, and this suggests that NPs can be used as a pesticide for target delivery (12, 56).

### Pesticide Delivery System Using Nanoparticles as Nanocarriers

**Figure 5:**
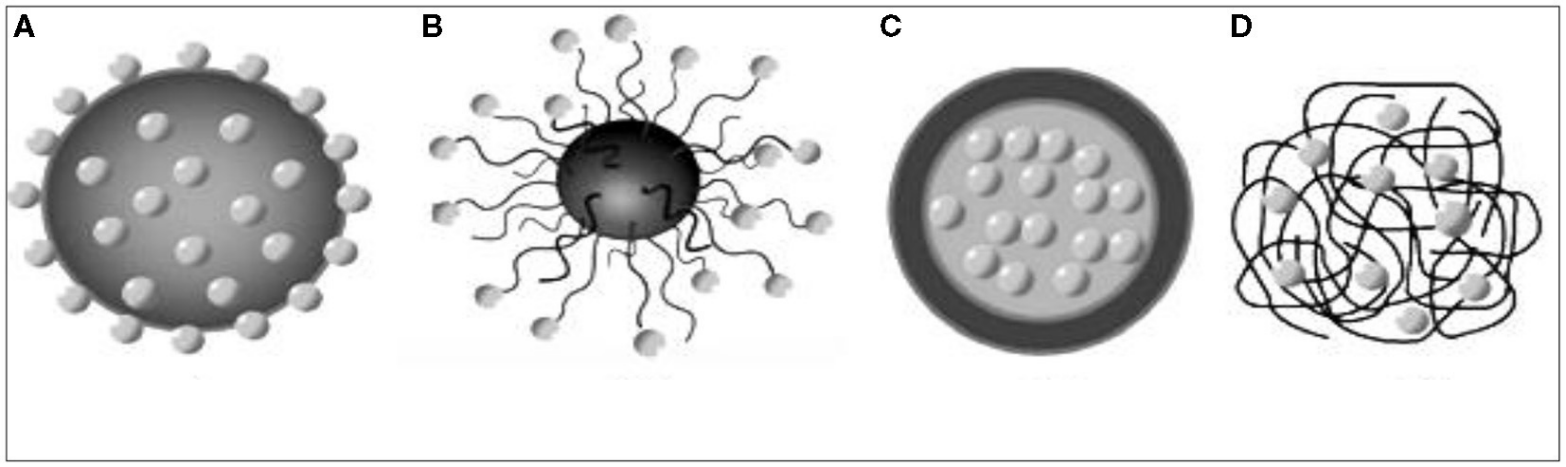
Schematic representation of NPs for delivery of pesticides, **(A)** adsorption on NP, **(B)** attachment on NP by different linkers; **(C)** encapsulation inside polymeric hydrophobic or hydrophilic core (polymer micelles); and **(D)** entrapment inside polymeric nanoparticle [source: Athanassiou et al. (10)].

The concept “pesticide delivery system” (PDS) was developed based on the drug delivery concept used in medicine where NPs are used to deliver the therapeutics to the target organs (61). PDS is designed to make available of the AI to a specific target at specified durations and concentrations to achieve the projected biological efficacy and by minimizing the harmful effects on non-target organisms (62). For the optimized release of sufficient and necessary amounts of pesticides within a specific time, controlled delivery plays an important role (61). Possession of specific characteristics of NPs, *viz*., high effective loading capacity, larger surface area, fast mass transfer to the insect's body (target), and the ability for easy attachment of various pesticide molecules, encouraged the use of NPs as nanocarriers. Pesticide molecules after encapsulations, show more gradual release over time, hence it requires fewer applications; at the same time, due to degradation, NPs hinder the loss ineffectiveness of the pesticides. For loading of pesticide molecules on NPs, different concepts, *viz*., encapsulation, adsorption, entrapment inside the NP, and covalent attachment reconciled by various ligands, are adopted (10) (Figure 5).

Based on the bonding of the AI to the material, degradation properties of the nanocarrier, and the environmental conditions, control and slow-release characteristics of the molecules could be attained. For the delivery of pesticides, nanocarriers like synthetic silica, polymer, titania, silver, alumina, and copper are mostly used. A few common examples of insecticides prepared using nanotechnology are listed in Tables 3, 4. Utilization of nanoencapsulation technique indicates safer usage of pesticides and less exposure to the ecosystem. Among all the NPs available, the solid and mesoporous silica NPs have shown the most potential delivery agent of agrochemicals due to their structural flexibilities for the formation of NPs of different shapes and sizes and ability to form pores during loading the biomolecules (58, 108–111).

**Table 3:** List of various encapsulated nanopesticides used against insect pest management.

**Nature of synthesized nanoparticles**	**Name of ingredients**	**Target organisms**	**References**
Synthetic	Avermectin	*Martianus dermestoides*	(63)
	Nanohexaconazole, Nanosulfur	-	(64)
	Nanopermethrin	*Cx. Quinquefasciatus*	(65)
	Imidacloprid	*M. dermestoides*	(66)
	Diethylphenylacetamide	*Culex tritaeniorhynchus*	(67)
	Nanopermethrin	*Aedes aegypti*	(68)
	Imidacloprid	-	(69)
	Temephos, Imidacloprid	*Cx. quinquefasciatus*	(70)
	Temephos,	*An. stephensi*,	(71)
Plant	*Moringa oleifera seeds*	*Stegomya aegypti*	(72)
	*Cuscutareflexa*	*Cx. quinquefasciatus*	(73)
	Plant oils	*-*	(74)
	*Copaifera* sp. *Oleoresin*	*A. aegypti*	(75)
	*Lippia sidiodes oil*	*A. aegypti*	(76)
	*Azadirachta indica* (Oil)	*Cx. quinquefasciatus*	(77)
	*Artemisia arborescens* (Oil)	*Bemisiatabaci*	(78)
	Garlic Oil	*Tribolium castaneum*	(79)
	*Azadirachta indica* (Oil)	*Bemisia tabaci*	(80)
	*Cuscutareflexa*	*Cx. quinquefasciatus*	(81)
Microbial	*Aspergillus flavus*,	*An. stephensi*,	(81)
	*Bacillus sphaericus*	*Cx. quinquefasciatus*	(82)

**Table 4:** List of various non-encapsulated nanopesticides used against insect pest management.

**Nature of synthesized nanoparticles**	**Name of ingredients**	**Target organisms**	**References**
Synthetic	Silver and Gold nanoparticles	*Aphis nerii*	(83)
	Silver, Aluminum Oxide, Zinc Oxide and Titanium dioxide Nanoparticles	insect pests and pathogens	(84)
	Nanosilica	*An. stephensi, Cx. quinquefasciatus A. aegypti*	(85, 86)
	Nanoalumina	*Sitophilus oryzae* (L.), *Rhyzopertha dominica* (F.)	(87)
Plant	*Nelumbo nucifera*	*Anopheles stephensi, Cx. quinquefasciatus*	(88)
	*Aloe vera*	*Anopheles stephensi*	(89)
	*Mukia maderaspatana*	*Cx. quinquefasciatus, A. aegypti*	(90)
	*Plumeria rubra*	*A. aegypti A. stephensi*	(91)
	*Jatropha gossypifolia, Euphorbia tirucalli, Pedilanthus tithymaloides, Alstonia macrophylla*	*A. aegypti*	(92)
	*Emblica officinalis*	*-*	(93)
Microbial	*Aspergillus*	*-*	(94)
	*Aspergillus*	*-*	(95)
	*Aspergillus*		(96)
	*Phytopthora infestans*	*-*	(97)
	*Aspergillus*	*-*	(98)
	*Aspergillus*	*-*	(99)
	*Trichoderma reesei*	*-*	(100)
	*Chrysosporium tropicum*	*Anopheles stephensi Cx. quinquefasciatus*	(101)
	*Epicoccum nigrum*	Pathogenic fungi	(102)
	*Aspergillus*	-	(103)
	*Rhizopus*	-	(104, 105)
	*Aspergillus niger*	*An. stephensi, Cx. quinquefasciatus, A. aegypti*	(106)
	*Schizophyllum*	*-*	(107)

### Polymer Nanoparticles as Nanocarriers

Polymer NPs that include polymeric nanocapsules and nanospheres are being used as polymer nanocarriers due to their flexibility to design a complex pesticide delivery system with various modes of actions, biocompatibility, scalable preparation, and biodegradation. In polymer nanospheres, the AIs of pesticide molecules (hydrophobic molecules) are randomly distributed with a core-shell structure in a polymer matrix in nanocapsules (known as polymer micelles), and this acts as a reservoir for encapsulation (112). Polymer nanocapsules provide advantages over larger capsules due to their improved uptake, the stability of the spraying solution, the spraying surface, and the homogenous distribution, which provides better knockdown of pests. As essential oils and secondary metabolites (phytochemicals) display stability problems (rapid evaporation and degradation of AI in presence of high temperature, moisture, and air) during the application, polymer nanocarriers can play a protective function and can increase their cost-effectiveness by maintaining the minimum effective dosage (62). For designing polymer formulations, different types of polymers, *viz*., polysaccharides (chitosan, starch, and alginates), polyesters [PEC (poly-e-caprolactone) and PEG (polyethylene glycol)], and biologically originated biodegradable materials (beeswax, cashew gum, lecithin, and corn oil) have been evaluated (113, 114). Among all of these polymers, PEG-based amphiphilic copolymers show better biodegradability, which is also easy for processing (112, 115). PEG polymer nanoformulation-based pesticides showed significantly slower release of the AIs in comparison to chemical pesticides like thiamethoxam (116), imidacloprid (117), beta-cyfluthrin (118), and carbofuran (119) and better efficacy than the chemical pesticides for control of nematodes (119, 120).

### Inorganic Nanoparticles as Nanocarriers

During the last two decades, for the formulation of pharmaceuticals, solid inorganic NPs are being used due to their various advantages *viz*., nanoemulsions, polymer NPs, better stability, higher loading, more controlled release, and low-cost production. Similarly, the solid inorganic NPs are used nowadays in agrochemical industries for the development of advanced pesticide delivery systems (14, 42, 62, 121–126). Among all the inorganic NPs, silica NPs are used mostly as nanocarriers for efficient delivery of biopesticides, insecticides, pheromones, and fungicides (49, 85). Silicon enhances the plant tolerance to different biotic and abiotic stress, and silica NPs are thus a potential candidate for use in pesticides for controlling agricultural pests (85), including tea pests. Pesticidal formulations based on silica NPs showed slow release of chlorfenapyr and promising results as an insecticide (127, 128). Under field conditions, the insecticidal activity of chlorfenapyr associated with silica NPs was found to be two times higher than the chlorfenapyr without NPs (128). To control insect pests during grain storage and field conditions, the prospective use of nano-silica has been reported against many pests (129–133).

### Using NPs Alone as Pesticides

NPs with insecticidal properties can be used not only as nanocarriers but also as biopesticides or active agents for pesticides (85, 134). The amorphous nano silica acquired from the shell wall of phytoplankton, vegetables, rice hulls, and volcanic soil is the most promising example of NPs with insecticidal properties (85, 135, 136). Similar to the mode of action of diatom particles (used for stored grain protection), the silica NPs can disrupt the protective barrier of insects and finally lead to the death of the same (85, 135, 137, 138). Earlier studies reported that the application of silica NPs in adult rice weevils, *Sitophilus oryzae*, caused 100% mortality (129). Similarly, a wide range of insect pests in agricultural and animal ectoparasites was successfully controlled using surface-charged modified hydrophobic silica NPs (139). The application of silica NPs as a thin film on seeds showed a reduction of fungal growth; on the other hand, it boosted germination (140). World Health Organization (WHO) considered safe for humans in the exploitation of silica NPs as a nano-bio-pesticide (138). Hence, silica NPs could be used as a pesticide for the control of agricultural and household pests, parasites, and fungi.

### Silver and Other Mineral-Based Nanoparticles

Similar tothe other nanomaterials, the metal-based NPs can be combined with pesticide molecules, which increases the effectiveness of the pesticide formulation (141–143) and reduces the application dose (144) for management of tea pests. These NPs can be synthesized solely using chemical compounds or involving living organisms (145). NPs based on silver, nanostructured alumina, aluminum oxide, zinc oxide, and titanium oxide have shown insecticidal properties. The larvae of case-bearing cloth moths, *Tinea pellionella*, showed 100% mortality while treated with ethanol-based nanosilver colloid (<20 ppm) (146). The wettable dust formulation of nanostructured alumina showed more than 95% mortality of *S. oryzae* and *R. dominica* adults infesting wheat within 3 days of exposure (87). Moreover, by combustion of aluminum nitrate and glycine, the prepared nanostructured alumina at 60 ppm dose showed more than 94% mortality for *S. oryzae* adults when applied as dust on wheat (147). Buteler et al. (148) obtained similar results for control of *S. oryzae* and *R. dominica* in wheat when novel nanostructured alumina was prepared based on chemical solution method and applied as dust. Buteler et al. (148) while studying the mode of action of the nanostructured alumina dust, reported that, through capillarity mechanism, the epicuticular lipids absorb the dust, which leads to dehydration of the insect and finally causes death. However, the effectiveness of the metal-based NPs depends on their physical properties, (*viz*., morphology, size, and surface area of the particle), biotic factors (*viz*., target species, exposure interval, and dose), and abiotic factors (relative humidity) (147, 148).

### Nano Bio-Pesticides

The selection of nano bio-pesticides have various important points for consideration, *viz*., they should be easy to prepare, economically viable, effective against a wide range of pests, must be safer to non-target organisms as well as the environment, non-toxic, should not accumulate in the food chain, should have nil or negligible residues, should not affect the quality of food, fragrance, texture, and flavor, and should be easy availability for application (149). Generally, for improvement of efficacy, better solubility, a slower rate of release and degradation, the traditional methods for the preparation of synthetic pesticides are blended (150). Similarly, for the formation of nano bio-pesticides, the biological compounds having pesticidal properties serve as capping and reducing agents and are blended with silver salt (149). Biologically synthesized NPs differ from chemically synthesized NPs in terms of their activities and effects on insect pests and plants. Due to the large surface area present in NPs, they can bond other compounds quickly and can circulate easily in the lepidopteran insect's system (85). Studies reported that nanocarrier materials with plant secondary metabolites cause indigestion, collapse the water protection barrier ultimately resulting in the death of insects (150). The nanosize of the compounds facilitates effectiveness with us a minimal quantity of bio-pesticides, protect the active secondary metabolites besides supporting the controlled release of the compound (150). The combination of a certain plant extract with nano-silica significantly increased the insecticidal activity as well as the shelf life (151). Most of the terpene compounds are reported to have antifeedant activity. Nanoformulation prepared using terpene compounds (alpha-pinene and linalool) by combining with nano-silica not only increases the efficacy (antifeedant activity against *S. litura* and *A. Janata*) but also enhances shelf life by more than 6 months (151, 152). Against tea pests, the nano bio-pesticides can be used both directly and indirectly as vectors as they can inherit some of the essential properties *viz*., permeability, stiffness, crystallinity, biodegradability, and thermal stability, and these properties are advantageous over commonly used synthetic pesticides. When AgNPs biosynthesized from the medicinal plant *Piper betle* leaf extract are tested against *Daphnia magna* for ecotoxicological studies, they showed less toxicity when compared to the chemically synthesized AgNPs, which confirmed the fact that biologically synthesized AgNPs are safer (because of the formation of the protein core around the NPs during synthesis) than the chemically synthesized AgNPs (153). Similar eco-friendly characteristics were reported with platinum (Pt)- and palladium (Pd)-biosynthesized NPs using *P. betle* leaf extract (154).

### Advantages of the Use of Nano-Pesticides Over Conventional Pesticides

To develop novel eco-friendly formulations for pest control, nanotechnology offers a tool for target-specific nanopesticides. Usually, target-specific nanopesticides could improve the effectiveness of pesticides and reduce pollution and undesirable residues in tea. They have slow release and protection performance as nanopesticides are prepared using thermo-sensitive, light-sensitive, humidity-sensitive, soil pH-sensitive, and enzyme-sensitive high polymer materials to deliver the pesticides. On plant surfaces, these formulations improve the adhesion of droplets, conferring improvement of dispersion and bioactivity of AI of target pesticide molecules. For these reasons, compared to conventional pesticides, nanopesticides show better efficacy for the management of tea pests. Nanopestides offer competent and eco-friendly advantages because of their small sizes, their efficacy when spraying in the field improvement of droplet adhesion on the plant surface, their wettability, as well as their rapid absorption by the target. It reduces the quantity of chemical usage, enhances plant protection, and reduces the toxic residues; hence nanopesticides enable the sustainable production of tea (155).

### Types of Nanopesticides

Nanopesticides play a major role in minimizing the footprints left by synthetic pesticides in agroecosystems. Encapsulated nanopesticides (Table 1) and several other nano-forms of silica, silver, iron, copper, and carbon (Table 2) are already in the practice of insect pest management.

### Potential of Nano-Pesticides for Agricultural Pest Control

Metabolites (terpenoids, flavonoids, and alkaloids) are plant-derived products (PDP) that consist of a complex mixture of compounds that act in diverse ways on insects, such as repellents, antifeedants, insect growth regulators, oviposition deterrents, and in terms of toxicity, and their mechanisms of actions are diverse (156). Most of the findings on the botanicals or PDP are restricted to the laboratory and have not been validated at field conditions (157). Indeed, marginal and small farmers in developing countries have introduced and regularly using the locally available plant products (water extract, crude oils, or seed cakes, etc) and commercial formulations [emulsifiable concentrations, wettable granules, solvent-based products, active ingredient (AI)] (156). These PDPs are established as effective commercial formulations (158). The presence of AI and allelochemicals in essential oils (EO) shows better efficacy than the PDP extracts (159). As a result of the quick “knockdown effect” on insect life stages and the accessibility of ready-made formulations, farmers use chemical pesticides in agriculture, but in most cases, these pesticides do not reach the target insect and are lost in the air, leached out in soil and water (62, 160). Besides, overuse or misuse of doses without following proper recommended mixtures and repeated applications of chemicals pesticides lead to several issues *viz*., development of insect resistance and undesirable residues in commodities and can harm humans and non-target organisms as well as the environment. Exposure to sunlight would lead to quick degradation of PDP and leave less persistence in the environment, have less of an impact on non-target organisms, and shows less residue (156). In comparison to the conventional chemical pesticides, the better properties in PDP nanotechnology are endorsed to controlled systems discharging small-sized molecules at the place of action, decreasing the residual problem, maximizing the action of the AI, increasing the target specificity, and increasing both the physicochemical stability and effectiveness of AI (62, 161). Nanopesticides are recognized to have less of an impact on human health and the environment and display less toxicity to non-target organisms as well as animals (161).

### Current Issues and Challenges

#### Bio-Efficacy

With the limited bioavailability of nanocarriers, both nanocarriers and AI need to be endowed with a requisite amount for attaining the preferred level of tea pest control. Hence, the durability and bioavailability of these components in PDP-based nanopesticides should be established (43). Commercial products, specifically the powder formulations confirmed better UV stability than the encapsulated nanoformulations (162). In practice, liquid formulations are preferred by the farmers. Hence, escalating persistence and stability in solid formulations is a challenge to the manufacturers. For this, Vrignaud et al. (163) suggested encapsulation of hydrophilic molecules in polymer-based nanoparticles and assessment of risks to non-target organisms with increasing uptake of AI to enhance the bio-efficacy of the same.

#### Toxicity to Plants

Azadirachtins in nanoformulation cause genotoxic and cytotoxic effects in plants, but during plant development, the toxicity is reduced to an undetectable level under sunlight (164). In another case, Liu and Xing (165) discussed different types of nanomaterials (nanotubes, Al, ZnO, Zn, and multi-walled carbon) that did not show any adverse effect on five different vegetables and ryegrass during seed germination and termination of root elongation. For corn plants treated with oleic acid and nanocapsules separately, Psquoto-Stgliiani (161) observed a negative effect on the physiological parameters against oleic acid, while there was no phytotoxic effect for nanocapsules.

#### Toxicity to Non-target Organisms in Agro-Ecosystem

Neem oil nanoformulation showed less toxicity to parasitoids of whitefly *Bemisia tabaci* and a parasitic wasp, *Encarsia formosa* when neem oil was added on polymeric nanocarriers (beta-cyclodextrin and poly-ε-caprolactone) (80). The toxicity of plant-derived metallic nanoparticles on microorganisms and earthworms depends on the concentration, particle size, number, and distribution of nanopesticides bound to the active ingredient (166). It is still debatable whether the release of metallic nanoparticles by microorganisms into the terrestrial environment is due to various effects of plant-derived metallic nanoparticles on the metalcore and capped phytochemicals (167). While Psquoto-Stgliiani et al. (161) confirmed that nanocapsules containing poly-ε-caprolactone treated with neem oil did not affect the soil microbes up to 300 days after exposure. Moreover, soils treated with nanoformulations enhanced the biomass of earthworms even up to 3 months of exposure to the formulation (168). Engineered nanomaterials can encourage surface coating and surface charge alongside other characteristics of physicochemical properties in the aquatic environment (167). As the nanopesticides may not be eco-friendly, it is necessary to understand the environmental fate and the role of surface capped plant-derived molecules in nano-specific processes (167).

#### Toxicity to Animals

Nanomaterials are safe at optimum size, but as they approach nanosizes, they often become toxic, especially the non-biodegradable materials (mostly metals). By accumulating and stimulating the immune system, they can cause toxicity in mammals, plants, and soils. Moreover, they can alter the activity of detoxifying enzymes (169). Aluminosilicate used in nanotubes can stick to leaf surfaces and can increase the stability of the product, even as the ingredients are also able to stick to the body surface of the insect pest, especially in the hairs, and can in due course enter the animal's body and affects physical functions (170). Apart from direct intake, dermal contact or inhalation can also lead to nanoparticles entering the human body. In pest control operators, chronic exposure and sub-acute causes inhalation toxicity, which is very common, and sometimes this leads to major health risks.

## Future Perspectives

For evaluation of the threat to the environment, existing regulatory protocols are pertinent only to synthetic insecticides used for controlling tea pests. As nanoformulations have a range of properties, consequently, there is a need to plan the guidelines for the nanopesticides (171). The risk assessment framework for future applications is to be accomplished thoroughly, as in the case of synthetic pesticides the experiments used for bulk production for commercial purposes may not hold valid for nanopesticides (172). Further study on the ratio of cost/benefit, price of the nanopesticides, recognition by the large scale as well as small tea growers, techniques for safe and easy application, availability, etc. would be ideal and can be achieved through further recommending the nanopesticides for management of tea pests.

Several research findings on various agricultural crop pests showed extensive confidence that nano pesticide-based formulations *viz*., nano-dispersions, nano-emulsions, and nanoparticles have potential and a bright future in terms of developing more effective and safer pesticide/biopesticide formulations for controlling tea pests. Conversely, this development will go through a sturdy inspection by national and international safety regulators because of the probable toxicity concerns of nanopesticides, which are not well-understood and are not standardized yet nor explored. During synthesis, changes in methods can cause changes in shape and dimensions, and the use of such materials is associated with risks. The process of synthesis of nanomaterials is thus so important. Consequently, before the use of those materials, studies related to the risk assessment are essential, as there are no definite guidelines to use these formulations on nanomaterials, nature of toxicity of those compounds to insects and plants needs to be explored. Before nanopesticides become popular in the tea sector for controlling pests, thorough research is needed by combining analytical techniques that can characterize the surface, detect the size, nature, or shape, and quantify the adjuvant and active ingredients emanating from the formulations. By reducing the number of chemicals for tea pest management, nanopesticides will make tea production profitable and eco-friendly. Although the progress in research and development is very slow, there seems to be a promising future for nanopesticides for tea pest management.

## Conclusions

Substantial crop loss occurs annually due to several notorious pests, including insects, mites, and nematodes. Tea, as a perennial monoculture crop cultivated in favorable climatic conditions, has become an ideal haven for a large number of insects and mite pests, diseases, and weeds, and this needs to be managed to avoid huge crop loss. Effective use of Nanopesticides for plant protection is an emerging research field and offers new methods to design active ingredients amid nanoscale dimensions in addition to the benefits of their formulation and delivery. Nanopesticide-based formulations *viz*., nanodispersions, nano-emulsions, and nanoparticles have potential and have a bright future in the development of more effective and safer pesticide/biopesticide formulations for controlling tea pests. There seem to be intense prospects for the effective use of nanopesticides for tea pest management.

## Data Availability Statement

The original contributions presented in the study are included in the article/supplementary material, further inquiries can be directed to the corresponding author/s.

## Author Contributions

BD prepared the draft manuscript. AB and MB edited the manuscript. CB assisted during preparing the manuscript. All authors contributed to the article and approved the submitted version.

## Conflict of Interest

The authors declare that the research was conducted in the absence of any commercial or financial relationships that could be construed as a potential conflict of interest.

## Publisher's Note

All claims expressed in this article are solely those of the authors and do not necessarily represent those of their affiliated organizations, or those of the publisher, the editors and the reviewers. Any product that may be evaluated in this article, or claim that may be made by its manufacturer, is not guaranteed or endorsed by the publisher.
